# The marriage of chemokines and galectins as functional heterodimers

**DOI:** 10.1007/s00018-021-04010-6

**Published:** 2021-11-12

**Authors:** Philipp von Hundelshausen, Kanin Wichapong, Hans-Joachim Gabius, Kevin H. Mayo

**Affiliations:** 1 Institute for Cardiovascular Prevention (IPEK), Ludwig-Maximilians-University Munich, 80336 Munich, Germany; 2 Department of Biochemistry, Cardiovascular Research Institute Maastricht (CARIM), Maastricht University, 6229ER Maastricht, The Netherlands; 3 Institute of Physiological Chemistry, Faculty of Veterinary Medicine, Ludwig-Maximilians-University, Munich, Germany; 4 Department of Biochemistry, Molecular Biology and Biophysics, University of Minnesota Health Sciences Center, Minneapolis, MN 55455 USA; 5 German Center for Cardiovascular Research (DZHK), Partner Site Munich Heart Alliance, Munich, Germany

**Keywords:** Cytokines, Glycoprotein, Inflammation, Lectin, Leukocytes

## Abstract

Trafficking of leukocytes and their local activity profile are of pivotal importance for many (patho)physiological processes. Fittingly, microenvironments are complex by nature, with multiple mediators originating from diverse cell types and playing roles in an intimately regulated manner. To dissect aspects of this complexity, effectors are initially identified and structurally characterized, thus prompting familial classification and establishing foci of research activity. In this regard, chemokines present themselves as role models to illustrate the diversification and fine-tuning of inflammatory processes. This in turn discloses the interplay among chemokines, their cell receptors and cognate glycosaminoglycans, as well as their capacity to engage in new molecular interactions that form hetero-oligomers between themselves and other classes of effector molecules. The growing realization of versatility of adhesion/growth-regulatory galectins that bind to glycans and proteins and their presence at sites of inflammation led to testing the hypothesis that chemokines and galectins can interact with each other by protein–protein interactions. In this review, we present some background on chemokines and galectins, as well as experimental validation of this chemokine–galectin heterodimer concept exemplified with CXCL12 and galectin-3 as proof-of-principle, as well as sketch out some emerging perspectives in this arena.

## Introduction

In order for single cells to form tissues and multicellular organisms, diverse types of “molecular glues” are required. On the biochemical level, homo- and heterotypic modes of recognition, along with the broad diversity of contacts with the extracellular matrix, bring cells together by a “cell adhesion code”. Structural characterization of glycoproteins involved in this complex process has provided direction to shape the nomenclature for its components. For example, the term “integrin” was coined “for the ‘integral’ membrane protein complex linking the extracellular matrix to the cytoskeleton” [1]. Paradigmatically, the structural theme (i.e. the fold and sequence signature that makes interactions possible) leads to diversity in molecular ancestry such that families of recognition molecules have emerged [2–4]. In parallel with this diversification, information coding that underlies pattern recognition has been extended beyond a discussion of the complementarity between proteins.

Sugars of cellular glycoconjugates are now also being recognized as biochemical messages. Glycans that present docking sites for receptors (lectins) store information within a minimal space (a specific set of spatially related amino acid residues within the folded protein) and thus are ideal for serving as cell surface signals [5, 6]. Protein–protein and protein–glycan interactions cooperate to ensure mechanical stability when building specific patterns. Of note, combinatorial permutations allow a glycoprotein (e.g. an integrin or extracellular matrix molecule like laminin or a lectican) to become involved in these types of molecular recognition that increase their applicability as cell biological tools. The engagement of integrins in such pairing covers the range from static to transient interactions, along with the ability to transmit outside-in signals [7, 8]. Along this route, integrin binding to a counter-receptor can account for modulating metabolic and transcriptional activities, apoptosis and growth, as well as for secretion of soluble mediators. These factors bring in a second and equally crucial means for cells to convey information. In principle, such secreted factors are essential for inter-cellular communication (without direct contact between surfaces). Here, we have used inflammation as a model system.

Considering the coordinated steps that allow leukocytes to home in on inflamed tissue, there is a distinct class of soluble factors that guides cellular movements within the trans-endothelial migration phase, i.e. the chemokines [9–11]. In fact, these small proteins are mediators of leukocyte recruitment [12], a starting point for thorough analysis of each family member and delineation of fundamental principles as expressed in the following statement: “Although many of the adhesion molecules and chemokines that direct leukocyte trafficking have been identified, there is still much to be discovered, particularly with regard to the persistence of leukocyte infiltration in chronic inflammation” [13]. Knowing what occurs among/between classes of various mediators may endow the microenvironment with hitherto unsuspected paired proteins with functional significance.

Apart from chemokines, one class of mediators that stands out from the population of regulators is the family of ga(lactoside-binding)lectins, i.e. galectins [14]. These mediators have been denoted as “exquisite modulators of the immune response” [13]. As broad-spectrum effectors of leukocyte activity and migration [15–17], galectins can serve as bridging molecules between both cells and cell surface glycoconjugates to construct highly ordered lattices in the membrane [18–25] and to act as sensors of damage- and pathogen-associated molecular patterns [26–28]. In this regard, elucidating their roles in inflammation deserves attention.

Our studies on both chemokine and galectin mediator classes have already resulted in discovering that expression of galectins and chemokines is correlated by a galectin-dependent enhancement of chemokine production and secretion. This intriguing phenomenon has been observed in various cell types, e.g. monocytes [29] or activated pancreatic stellate cells (that secreted the chemokine CXCL12 promoting pancreatic cancer cell invasiveness) [30, 31], rheumatoid synovial fibroblasts [32], osteoarthritic chondrocytes [33, 34], endothelial cells [35, 36] and bone marrow-derived dendritic cells [37]. Learning more about these protein mediator families, their structures and interacting partners are the bases for assessing whether there is more than just a galectin-induced upregulation of chemokine expression that connects these families and makes both classes available in the microenvironment.

## Cell migration

**Fig. 1:**
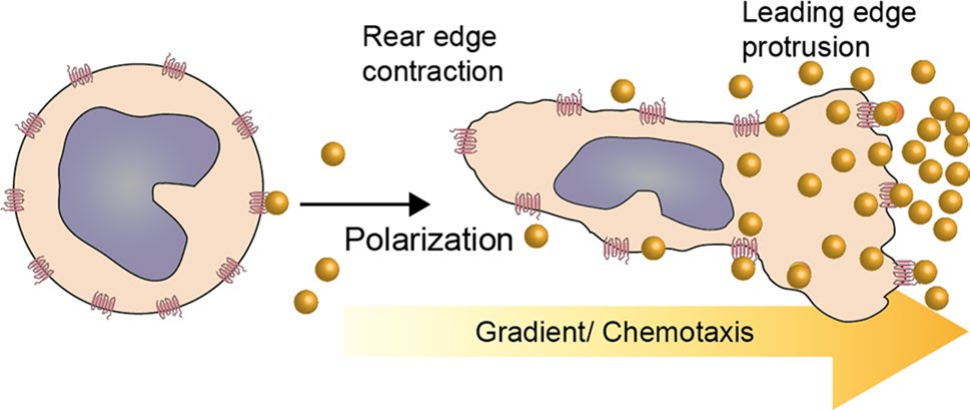
Cartoon depiction of leukocyte polarization and migration given direction by a chemotactic gradient

One cellular elicitor function that chemokines and galectins have in common is cell migration and activation. Positioning cells within a tissue at predestined sites is fundamental to physiology, e.g. in embryonic development, tissue regeneration, and host defense. In these instances, signals provide direction to cell motion via chemoattractants as sensed by their cell receptors. Cells are thereby directed to various destinations by following chemoattractant gradients. The absolute concentration and temporal dynamics of a gradient determine the velocity and persistence of migrating cells and other signaling molecules that affect these parameters by regulating receptor desensitization [38]. Once one side of a cell senses presence of a chemoattractant, the cell becomes polarized with this initial contact side (forming the leading edge for cell movement) with protrusions extending towards the direction of cell migration [39], as shown in Fig. 1.

When in contact with a surface, polarized cells interact with adhesion molecules, such as integrins. Their binding to counter-receptors at the cell surface generates traction forces that promote relatively rapid locomotion. Even without these molecular handles, leukocytes can move around. If confined in a three-dimensional environment, leukocytes use (in addition to or in the absence of adhesion molecules) friction to the topographical particularities of a solid matrix to migrate [40]. To do so, however, the rearrangement of the actin cytoskeleton nestles closely to the substratum, thus generating retrograde shear forces sufficient to propel the cell forward [40]. In the absence of a matrix (i.e. in solution), leukocytes swim using a breaststroke-like motion with cell protrusions which serve as paddles that are enriched in proteins [41].

Leukocytes routinely and randomly patrol cell surfaces and in tissues, an important surveillance that allows detection of pathogen invaders. It is assumed that cells ‘understand’ directional cues by sensing fields of chemoattractants, and it is via these processes that chemokines and galectins are the central mediators. Indeed in tissues, chemokines form gradients that enable haptotactic migration of leukocytes [42], and galectins (e.g. galectins-1, -3 and -8 (Gal-1, -3 and -8)) promote cell migration of immune cells [16], epithelial cells [43] and glioblastoma cells [44–46], respectively, as they can be suppressive in a context-dependent manner like Gal-8 with colon cancer cells [47].

## Chemokines

The genesis of the molecular era of chemokines is attributed to several findings, one being the discovery of platelet factor 4 (PF4 or CXCL4), a structural proto-type chemokine that was purified by exploiting its high affinity for heparin [48]. Functionally, PF4 differs from most other chemokines by a lack of a high-affinity G-Protein Coupled Receptor (GPCR) and a lack of chemotactic activity [48–50]. From a functional perspective, the first human chemotactic factor, MCP-1, was isolated from supernatants of activated leukocytes and thus designated to be a member of a family of lymphokines or intercrines [51, 52]. Following its discovery, a steadily growing number of related small proteins (~ 8 kDa) were identified and placed into the so-called PF4 super-family (for a survey of human chemokines, please see Table 1).

**Table 1:** Human chemokines, their quaternary structures and main receptors

Human chemokines	Systematic name	Conventional name(s)	Protein data bank ID*	Quaternary structures	Main typical and atypical chemokine receptors
CC family	CCL1	I-309; TCA3	1ELO, 4OIJ, 4OIK	CC dimer	CCR8, CCR11
	CCL2	MCP-1; MCAF	1DOK, 1DOL, 1DOM, 3IFD	CC dimer, **CXC dimer/tetramer**	CCR2, *ACKR1, ACKR2*
	CCL3	MIP-1α; LD78α	1B50, 2X69, 5COR, 5D65	CC dimer, polymer	CCR1,CCR5, *ACKR2*
	CCL3L1	LD78β	NA		CCR1, CCR5
	CCL4	MIP-1β	1HUM, 1JE4, 2X6L	CC dimer, polymer	CCR5, *ACKR2*
	CCL4L1	LAG-1	NA		CCR5
	CCL5	RANTES	1HRJ, 1RTN, 1U4L, 2L9H, 5CMD, 5L2U, 6AEZ, 6C6D, 6STK	CC dimer, trimer, hexamer, 20-mer	CCR1, CCR3, CCR4, CCR5, *ACKR1, ACKR2*
	CCL7	MCP-3	1BO0, 1NCV	Monomer > **CXC dimer**	CCR1, CCR2, CCR3, *ACKR1, ACKR2*
	CCL8	MCP-2	1ESR	CC dimer	CCR1, CCR2, CCR5, CCR11, *ACKR2*
	CCL11	Eotaxin	1EOT, 2EOT, 2MPM	Monomer > CC dimer	CCR3, *ACKR1, ACKR2*
	CCL13	MCP-4	2RA4	CC dimer	CCR1, CCR2, CCR3, CCR11, *ACKR1, ACKR2*
	CCL14	HCC-1	2Q8R, 2Q8T	CC dimer	CCR1, *ACKR1, ACKR2*
	CCL15	HCC-2	2HCC	Monomer	CCR1, CCR3
	CCL16	HCC-4; LEC	5LTL	CC dimer	CCR1
	CCL17	TARC	1NR2, 1NR4, 5WK3	CC dimer, tetramer	CCR4, *ACKR1, ACKR2*
	CCL18	PARC; DC-CK1; MIP-4	4MHE	Monomer > CC dimer	CCR8
	CCL19	MIP-3β; ELC	2MP1	Monomer	CCR7, *ACKR4, ACKR5*
	CCL20	MIP-3α; LARC	1M8A, 2HCI, 2JYO,5UR7, 6WWZ	**CXC dimer**	CCR6
	CCL21	6Ckine; SLC	2L4N, 5EKI	Monomer, hexamer	CCR7, *ACKR4*
	CCL22	MDC; STCP-1	NA	NA	CCR4, *ACKR2*
	CCL23	CKβ8; MPIF-1	1G91	Monomer	CCR1
	CCL24	Eotaxin-2; MPIF-2	1EIG	Monomer	CCR3
	CCL25	TECK	NA	NA	CCR9, *ACKR4*
	CCL26	Eotaxin-3	1G2S, 1G2T	Monomer	CCR3
	CCL27	CTACK; ILC; ESKINE	2KUM	Mixed CC and CXC dimers, tetramer	CCR2, CCR3, CCR10
	CCL28	MEC	6CWS	Monomer	CCR3, CCR10
CXC family	CXCL1	GRO-α; MGSA-α; MIP-2	1MGS	CXC dimer	CXCR2, CXCR1
	CXCL2	GRO-β; MGSA-β; MIP-2α	1QNK	CXC dimer	CXCR2
	CXCL3	GRO-γ, MGSA-γ; MIP-2β,	NA		CXCR2
	CXCL4	PF4	1RHP, 4R9W	CXC dimer, tetramer	CXCR3
	CXCL4L1	PF4alt; CXCL4V1	4HSV	CXC dimer, tetramer	CXCR3
	CXCL5	ENA-78	2MGS	CXC dimer	CXCR1, CXCR2, *ACKR1*
	CXCL6	GCP-2	NA	NA	CXCR1, CXCR2, *ACKR1*
	CXCL7	PPBP; CTAP-III; β-TG; NAP-2	1F9P, 1NAP, 1TVX	CXC dimer, tetramer	CXCR2
	CXCL8	IL8	1IL8, 2IL8, 3IL8, 6LFM	CXC dimer	CXCR1, CXCR2, *ACKR1*
	CXCL9	MIG	NA		CXCR3
	CXCL10	IP-10; CRG-2	1VL9, 1O7Y	CXC-type dimer	CXCR3
	CXCL11	I-TAC,	1RJT	Remains monomeric	CXCR3, *ACKR3, ACKR1*
	CXCL12 α,β,γ	SDF-1 α,β,γ	1QG7, 1SDF, 1VMC, 2J7Z, 2KEC, 2SDF, 3HP3	CXC-type **and CC- type dimers**	CXCR4, *ACKR3*
	CXCL13	BCA-1; BLC	6VHJ, 7JNY	Atypical β0 CXC-type	CXCR3, CXCR5
	CXCL14	BRAK	2HDL	NA	Unknown
	CXCL16	SR-PSOX	NA		CXCR6
	CXCL17	DMC, VCC1	NA		Unknown, GPR35
XC family	XCL1	Lymphotactin; SCM-1α; ATAC	1J9O, 2JP1, 2NYZ	Alternative dimer	XCR1
	XCL2	SCM-1β	NA		XCR1
CX3C family	CX3CL1	Fractalkine; Neurotactin; ABCD-3	1F2L*	Unique, CC-like	CX3CR1
Atypical chemokines (ACK)*					
		MIF	1CA7, 1GCZ, 1GIF, 1MIF,	Monomer resembles CXC-type dimer, trimer barrel	CXCR2, CXCR4, *ACKR3*
		hBD-1	1E4S, 1IJU, 2NLB	Monomer	CCR6
		hBD-2	1FD3	Non chemokine like dimers, octamer	CCR2
		hBD-3	1KJ6	Not well defined dimer	CXCR4, CCR6, CCR2
		hBD-6	2LWL	Monomer	CCR2

Quaternary structures of CC chemokines that can form CXC-type dimers and vice versa are highlighted bold. ACKR1 (DARC, Duffy Antigen Receptor), ACKR2 (D6). ACKR3 (CXCR7), ACKR4 (CCRL1), ACKR5 (CCRL2)

*PDB entries of wild-type chemokines were taken from rcsb.org if a corresponding publication was available

Chemokine classification is based on the position of a highly conserved cysteine motif within the N-terminal sequence of the protein. In the two largest classes of chemokine, this motif consists of two cysteine residues either separated by a single variable amino acid residue (CXC family) or placed in tandem (CC family or RANTES/SIS family) [53]. To avoid the use of several names for this family of chemotactic cytokines, the “Third International Symposium on Chemotactic Cytokines” held in 1992 in Baden (Austria) established the generic term “chemokine” [54].

From a genetics point of view, it was the gene of one of these small, inducible cytokines (I-309) that gave rise to the now commonly used gene name *SCYA1*, with *A* being a classifier for the CC family and with the gene for Gro-α introducing the B/CXC family (*SCYB1*). In both mouse and human, these gene families occur in several clusters on distinct chromosomes. At that time, the molecular details of chemokine receptors were just beginning to emerge, such that in 1997 an NC-IUPHAR subcommittee on chemokine receptor nomenclature developed the idea of numbering signaling receptors based on the gene number of the respective chemokine ligand [55]. Thus, the receptor being bound and activated by *SCYA1* coding for I-309 (now CCL1) became known as CCR1, and the receptor for *SCYB1* coding for Gro-α became known as CXCR1.

Members of the hepta-helical group of receptors that bind chemokines, but do not associate with G-proteins (such as the duffy antigen receptor DARC) were excluded and later called atypical chemokine receptors (ACKRs) [56]. Currently, the human system comprises CCR1-CCR10, CXCR1-CXCR6, CX3CR1, XCR1 and ACKR1-6 (CXCR7 = ACKR3). To unify the numerous synonymous names for chemokines, the nomenclature for chemokine receptors was adapted in 1998 to chemokines. For example, *SCYA1* encodes chemokine CCL1 (I-309) and *SCYB1* CXCL1 (Gro-α). In humans, the CC-branch comprises CCL1–CCL28, the CXC-branch CXCL1–CXCL17, whereas CX3CL1 and XCL1-2 are the only members of the X3 and C families, in which the N-terminal Cys residues are separated by three other residues or only a single N-terminal cysteine exists (Table 1). In addition, some effector proteins like β-defensins, (macrophage) migration inhibitory factor (MIF) and high-mobility group protein 1 (HMGB1) share structural and functional features with chemokines and have been dubbed atypical chemokines or non-chemokine agonists as they bind to chemokine receptors (Table 1) [57, 58].

## Chemokine structures

**Fig. 2:**
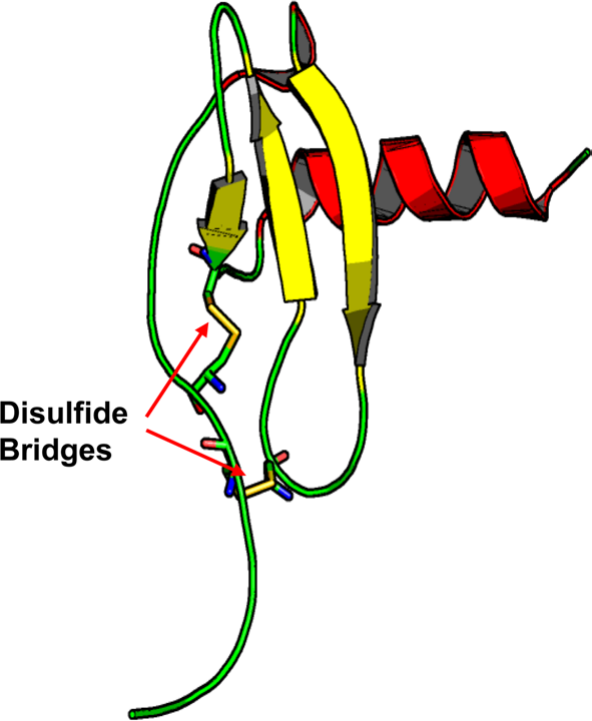
Monomer structure of CXCL12 (PDB code: 4UAI). β-Strands are shown in yellow, and the C-terminal α-helix is shown in red. The two cystine bridges are colored in green/yellow sticks, with red arrows indicating their positions

Chemokines have a characteristic tertiary structural fold known as the Greek key in which a dynamic N-terminal segment leads into a three-stranded β-sheet (β1, β2, β3), followed by a loop (the 50s loop) and a C-terminal α-helix that folds onto the β-sheet (Fig. 2). The two highly conserved cysteines at the N-terminus of any chemokine provide structural stability by forming disulfide bridges with two other strategically positioned cysteines, one within the 30s loop that links to the first N-terminal cysteine and a second cysteine between β-strands β1 and β2 that forms a disulfide bond with the sulfhydryl of the other N-terminal cysteine (Fig. 2).

**Fig. 3:**
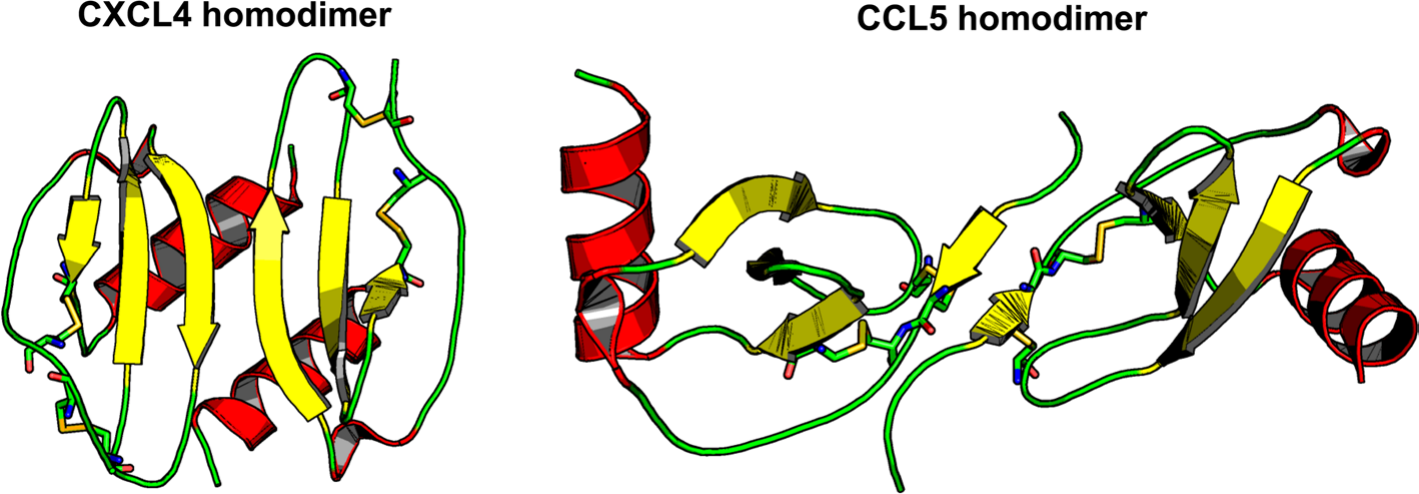
CXCL4 and CCL5 homodimers (PDB codes: 1F9Q and 6STK, respectively) are shown. Colors are displayed according to their secondary structures: β-strands in yellow, C-terminal a-helix in red, and loops in green. Cystine bridges are illustrated by yellow/green sticks

Most chemokines can associate as homodimers (and in some instances to higher-order oligomers like CXCL4 (PF4) that forms tetramers) [59, 60]. CXC and CC chemokines dimerize differently, yet in a conserved fashion. CC chemokines dimerize via interactions between N-terminal sequences from two individual monomers, whereas CXC chemokines dimerize by anti-parallel β-sheet formation between the β1-strands from two monomers, thus extending the 3-stranded β-sheet in each monomer into a 6-stranded β-sheet, as well as by anti-parallel interactions of their helices with the surface of the β-sheet [61], as shown in Fig. 3. There are some exceptions, however, namely CCL20 and CCL7 that actually form CXC-type dimers, XCL1 that forms a β-sandwich-type structure [62, 63], CXCL12, CCL2 and CCL27 that can exist in both CC- and CXC-type conformations (Table 1) [64–66].

When examined in vitro in solution, chemokine homodimer dissociation constants normally fall in the µM range [60, 67, 68]. Some chemokines in solution exhibit larger K_D_ values and thus exist primarily as monomers, as exemplified with CXCL7 [68] and LA-PF4 (low affinity PF4) [67]. Nevertheless, given their normally less than µM concentrations in plasma [69, 70], chemokines should be present as a distribution of monomers and homodimers in situ. Although under in vitro conditions chemokine oligomerization requires these relatively high concentrations, oligomerization has been shown to be relevant for leukocyte recruitment in vivo where much lower concentrations occur [71]. The explanation for this is that oligomerization depends on the chemical environment such as pH, presence of ions and importantly on the presence of glycosaminoglycans (GAGs) [72, 73]. The GAG contact region of the monomeric CC chemokines (CCL3, CCL4, CCL5) differs from that of their oligomer, because the oligomer conceals the BBXB recognition motif (B represents a basic residue) in the 40’s loop, instead another cluster of basic residues in the 50’s loop becomes the contact region, again illustrating the enormous complexity of the regulatory system [74]. Therefore, under steady-state conditions, chemokine dimerization is facilitated. Moreover, in inflamed tissues, local concentrations can be much greater, with their homodimeric state being significantly influenced by the overall cellular environment, e.g. local pH, binding partners, ligands, and hydrophobic vs. hydrophilic interactions, as exemplified with CXCL4 (PF4) under various solution conditions [75]. Therefore, dissociation constants for chemokine dimerization measured in vitro are likely to be higher than those from a given chemokine in a physiological environment [69].

Chemokine oligomerization has implications for, e.g. cell migration and signaling, and is required for full activity in vivo [71] (for reviews, see also [76, 77]). As mentioned above, the chemical environment can strongly influence the extent of dimerization. Consider CXCL12 where negative ions (e.g. phosphate, sulfate), increasing pH, addition of GAGs, as well as the presence of CXCR4-derived peptides, can promote its dimerization [72]. The ability of chemokines to oligomerize is considered an additional means of regulation of chemokine activity and, of note, gradient formation. This process is modulated by local microenvironmental factors. Both CC- and CXC chemokines can aggregate in solution and on cell surface-presented GAGs, with chemokine-specific differences being reported [76, 78]. Interestingly, the degree of sulfation and the positions of sulfate groups are key factors that regulate affinity, with this type of interaction being defined by some sort of sulfation code. How GAGs regulate chemokine function by affecting monomer/dimer levels, gradients, and importantly how GAGs may present chemokines to their receptors is a matter of ongoing research and been reviewed for ELR-chemokines binding to CXCR1 and/or CXCR2 [79].

**Fig. 4:**
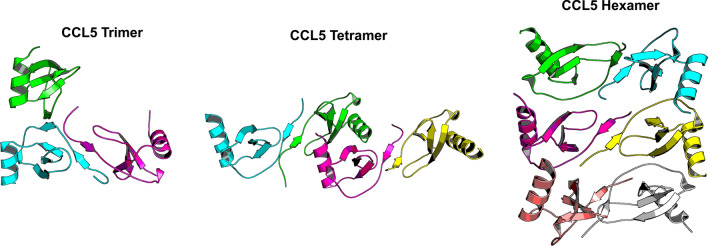
CCL5 monomers can associate into various oligomeric states. The structures shown are for the trimer (PDB code: 6AEZ), CCL5 tetramer (PDB code: 2L9H), and CCL5 hexamer (PDB code: 5CMD)

To address the question as to whether chemokine homodimers or monomers bind to and activate their cell receptors, chemokines that normally form dimers have been engineered to prefer the monomeric state by mutating residues within the interfacial β1 strand for CXC chemokines [80, 81] and within the interfacial N-terminal sequence of CC chemokines [71, 82]. Using these monomer-engineered variants, it has been shown that monomers can retain full receptor-activating capacity, suggesting that chemokines can bind and activate their receptors as monomers. In contrast, covalent (also named locked or trapped) dimers of CXCL8 and CXCL12 have also been engineered, and show differential effects on signaling. Whereas the CXCL8 dimer is a potent CXCR2 agonist [81, 83], it has attenuated effects on CXCR1 and likewise the locked CXCL12 dimer has differential effects opposed to its constitutive monomeric variant, suggestive of biased agonism [84]. With Cryo-electron microscopy it has been possible to elucidate the structure of a CXCL8 binding as monomer or dimer to CXCR2 [85]. Here, CXCL8 chain A binds with its N-terminus into the pocket and its core domain to the receptor N-terminus whereas CXCL8 chain B resides mainly piggy-back on CXCL8 chain A weakly interacting with extracellular loop 2 of CXCR2 [85]. This supports the biological significance of distinct quaternary structures. Some chemokines can also associate into higher-order oligomers, as illustrated by formation of CCL5 di-, tri-, tetra- and hexamers (Fig. 4) and polymers of CCL3 [86], a structural issue that has physiological relevance. [74, 86]

**Fig. 5:**
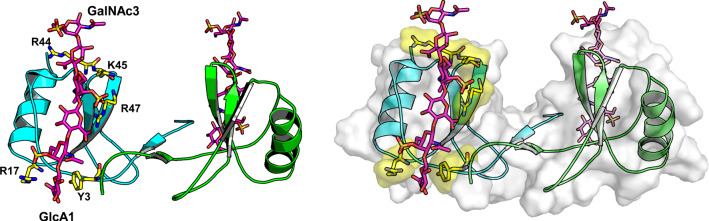
A model of CCL5 in complex with a chondroitin sulfate hexasaccharide (magenta sticks) is shown [184]. GAG binding residues R17, R44, K45 and R47 are highlighted in yellow sticks

Although chemokines bind GAG chains with high affinity, the resulting biological consequences are diverse and may even present opposing effects. To explain this apparent diversity of experimental outcome, the versatility for structural heterogeneity of cellular GAGs should be noted, i.e. GAG chain length as well as degree and position of sulfation, because these factors affect chemokine binding [87]. The binding event also depends on whether the GAG is in solution (e.g. injected heparin) or bound to, and thus presented by, proteoglycan core proteins on cell surfaces. Providing the quintessential case for chemokine heparin binding, CXCL4 (PF4) binds the GAG at specific sites via interactions with a cassette of four lysines in its C-terminal α-helix, and the loop containing Arg20, Arg22, His23 and Thr25 (as well as with Lys46 and Arg49), with the latter group actually being more crucial to GAG binding than lysines in the C-terminal helix [88]. With CXCL12, heparan sulfate interacts primarily with the homodimer interface, along with a cluster of basic amino acid on the exterior of the dimer [89]. GAG binding promotes formation of chemokine homodimers [71, 78, 90, 91], as well as affecting chemokine structure [90, 92]), structural dynamics [93], and chemokine dimerization [94]. In addition, heparin dodecasaccharide binding to CXCL4 induces higher-order oligomer formation that is dependent upon the chemokine GAG molar ratio, an effect that has implications for heparin-induced thrombocytopenia [95]. As a chemokine-binding partner, GAGs are pivotal to the broad spectrum of chemokine-mediated biological activities. In line with a crucial role for haptotaxis (i.e. chemotaxis along immobilized gradients), variants of CCL2, CCL4 and CCL5 that cannot bind GAGs cannot bind to the cell surface, and, although these three chemokines retain activity in vitro, they do not mediate leukocyte recruitment in vivo [71]. Further support for this concept stems from studies with the skin of mice where endogenous CCL21 gradients established on heparan sulfate have been visualized, these gradients enable directed migration of dendritic cells to lymphatic vessels [42] (Fig. 5).

Thus, while the importance of GAGs in chemokine recruitment and accumulation and gradient formation is well recognized, the role of GAGs in chemokine receptor activation is less well understood. There may be different mechanisms depending on the particular chemokine and its oligomeric state. If the sites where GAGs and receptors can bind to the chemokine overlap and interfere, ternary complex formation is impossible. For this case, the chemokine cloud model was presented by Graham et al. stating that “glycosaminoglycans provide an immobilized chemokine depot maintaining a ‘cloud’ of ‘solution-phase’ chemokines within the glycocalyx, and that it is this soluble form of any given chemokine that interacts with leukocyte-bound receptors” [96]. On the other hand, an alternative model for chemokines, specifically ELR-positive CXC chemokines such as CXCL8, was proposed by Rajarathnam et al., in which one chemokine binds to the GAG and the other to the receptor in a homodimer [79].

## Chemokine–chemokine heterodimers

Because the structure of all chemokine monomers is highly conserved, their ability to form homodimers is dictated primarily by residues at the inter-subunit dimer interface [97]. With this in mind, we hypothesized that monomers of different chemokines can exchange from one chemokine to another when the composition and spatial arrangement of residues at the heterodimer interface is energetically favorable vis-à-vis those in either chemokine homodimer [98]. For example, CXCL4, its N-terminal chimera PF4-M2 [88] and CXCL8, as well as CXCL1 and CXCL7, readily swap subunits to form heterodimers with K_D_ values similar to those of their respective homodimers [98–100].

Heterodimers of CXC and CC chemokines (e.g. CXCL4 and CCL5) also form in cultured cells, as well as in vivo [101, 102]. CC chemokines CCL3/4 and CCL2/8 (macrophage inflammatory protein-1α (MIP-1α) and -1β (MIP-1β), respectively, Table 1) can also form heterodimers in vitro, as well as being secreted as heterodimers from activated monocytes and blood lymphocytes [103]. The detectability of chemokine heterodimers under these conditions where chemokine concentrations are lower than those, e.g. in NMR studies, indicates that a physiological, cellular environment can facilitate homo- and hetero-dimerization. Indeed, the presence of GAGs increases chemokine heteromerization in such a way that in some instances, e.g. CCL2-CCL11 and CCL8-CCL11, the heterodimer is preferred over the homodimer [94]. As with chemokine homodimers, their heterodimers are also structurally stabilized by GAG binding [104, 105].The GAG contact sites in (hetero)oligomers can be different from those in the monomer. One example is CCL5 that interacts with its BBXB motif in the 40’s loop that becomes buried upon oligomerization and is exchanged by a motif of basic residues in the 50’s loop. This CCL5-CXL4 heterodimer can explain some functional effects, because they are protected from proteolytic degradation [74, 104]. Many questions (especially when it comes to multiple coexisting chemokines) remain open. For example, could it be that several chemokine gradients exist simultaneously or that multiple chemokines can assemble into higher-order structures? Moreover, it has been postulated that chemokine activity may be regulated through GAGs, independent of chemokine receptor binding [74].

There is compelling evidence that chemokine homo- and heterodimers can exert biological activity through chemokine receptors even if this activity is biased and deviates from the monomeric form. This was demonstrated using obligate (constitutive) forms that are covalently and specifically linked to mimic their natural counterparts. This has been shown for homodimers of CXCL1 [83], CXCL8 [81], and CXCL12 [84], as well as heterodimers CXCL7-CXCL1 [106] and CC-type CCL5-CXCL4, CCL5-CCL17 [102]. Their activity range expressed as EC50 values fall in the nanomolar range (5–15 nM). GAGs bind to a heterodimer in a way where one partner binds and supposedly recruits the chemokine to a GAG, whereas the other partner activates the receptor as shown for the heterodimer CXCL4-CCL5 with CCL5 being the active part [102]. An alternate explanation has been presented by Liang et al. who explain the activity of CCL5-CXCL4 heteromers with a model that shows the assembly of CCL5-CXCL4 heterodimers into higher-order heterooligomers with increased affinity for the GAG [74]. Another mechanism happens on cells where chemokine heterodimers meet heteromers of their receptors as shown for CCL5-CCL17 and CCR5-CCR4 on T cells [102].

**Fig. 6:**
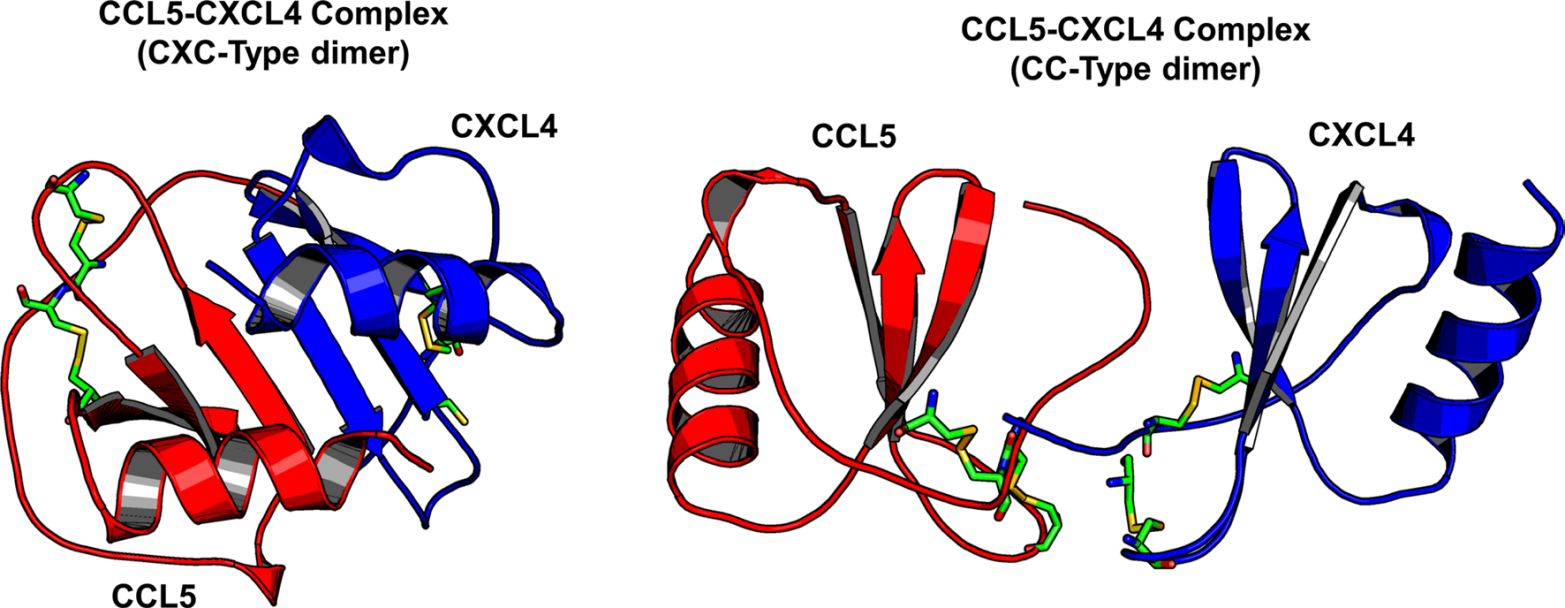
Models of the CXCL4/CCL5 heterodimer are shown. These structures are based on NMR spectroscopical HSQC chemical shift and intensity changes measured in a mixture of ^15^N-labeled CXCL4 and ^15^N-labeled CCL5. NMR data directed manual docking and energy minimization using MD simulations [102]. This in silico study was performed using CXCL4 and CCL5 monomers initially docked as a CXC-type dimer or a CC-type dimer as indicated. The CC-type heterodimer was highly energetically favored. CCL5 monomers are in red, CXCL4 monomers are in blue, and cystine bridges are colored in green/yellow sticks

Nesmelova et al. used molecular dynamics (MD) and mechanics simulations to calculate free energies for heterodimerization of several CXC and CC chemokines and reported that a number of CXC and CC chemokine pairs (within and between CXC and CC families) may associate as heterodimers in solution [97]. For example, CXCL4 forms CXC-type heterodimers with CXCL1, CXCL7, and CXCL8, as well as CXCL1/L8, CXCL7/L8 and CXCL1/L7. In addition, CCL2 can form CC-type heterodimers with CCL5 and CCL8, as well as CXC-type heterodimers with CCL8 [13, 97]. It seems plausible that when chemokines dimerize within an interaction type (CC, CXC) and exchange monomers, the interaction type is retained. However, there are cases, e.g. when chemokines of different types are combined, where it is more difficult to predict which heterodimer type (CXC or CC) will prevail. Typical examples are CCL5, which forms a CC-type heterodimer with CXCL4. Some mixed chemokine combinations, such as CCL2 and CXCL8, can give rise to both CC and CXC types. Surprisingly, there also seem to be heterodimers of two different CC chemokines, such as CCL2 and CCL8, which can form both CC- and CXC-type heterodimers [97]. Modeled CXC-type and CC-type heterodimer structures are shown in Fig. 6 for the CXCL4/CCL5 pair, with the CC-type heterodimer being more energetically favored, an observation that has been empirically validated in vitro and in vivo [102].

## Chemokine receptors

In most cases, chemokine signaling is mediated by conventional chemokine receptors. This is not the case when a chemokine has no high-affinity receptor as with CXCL4 that has an indirect effect on chemokine cell receptors. Instead of direct binding to a cognate chemokine receptor, CXCL4 primarily may exert its biologic activity by binding to and modulating the activity of other soluble mediators, including growth factors (FGF-2, VEGF) and chemokines as we will discuss in a later section [99, 107–110]. Due to a lack of stability, it has been impossible to delineate the three-dimensional structure of a wild-type chemokine receptor in complex with a soluble wild-type agonist chemokine by crystallography or NMR spectroscopy. However, in combination with ortho- and allosteric small molecule antagonists or using engineered variants that produce stable complexes, it has been possible to obtain structures of the G-Protein coupled receptors CXCR4, CCR2, CCR5 and CCR6 [111–114]. Only recently has cryo-EM made it possible to resolve the structure of a chemokine (CXCL8) in association with its receptor CXCR2 [85].

Most investigators assume a two- or three-step receptor-activation model in which the chemokine initiates the first binding step with the N-terminal domain of the receptor (site I) interacting with the dynamic N-terminus and N-loop [115]. Known structures of the receptor N-domain with the chemokine similarly show that the residues proximal to the second cysteine of the N-domain are involved [116]. Due to their low sequence homology, it is likely that it is the N-domains that confer binding specificity. The N-domain of CXCR1 and CXCR2 have been reported to reach out to and catch CXCL8 which happens with moderate affinity. This induces a subsequent stabilization of the receptor N-domain [85, 116] and leads to a subsequent movement of CXCL8 until a position on the receptor and a high-affinity conformation is reached where the chemokine interacts with site II so that the residues that mediate the contact change during the binding process. This conformation allows CXCL8 to bind to CXCR2 in both the monomeric and dimeric forms, as the dimer interface is not affected [85].

While the contact region of the N-domain is similar for the receptor structures we know, the corresponding contact region of the chemokines varies. For instance the N-domain of CCR5 binds to two regions, the N-loop and the BBXB motif in the 40s loop of CCL5 that is important for GAG binding whereas the N-domain of CXCR4 interacts with the β1-strand of CXCL12 [117]. CXCL12 has been engineered to be in either a constitutive monomeric or dimeric form and both forms bind differently to the CXCR4 N-domain with the monomer interacting with the residues of the most distal residues and the dimer preferring the proximal residues [118]. Whether ternary complexes of receptors and chemokine (hetero)dimers can possibly form depends on whether the contact regions on the chemokine for GAG, receptor and dimerization compete which needs to be investigated for each case in the future.

Superposition of these structures shows that the N-terminus of the chemokine ligand ‘dips’ into the receptor. However, major differences exist in the precise mode of binding or how deeply the chemokine dips into the binding pocket [112].

GPCRs adopt distinct conformations that are in dynamic equilibrium [119], and binding of a chemokine agonist to the extracellular portion of the receptor shifts that equilibrium to an activated state [119]. Chemokine receptors bind to several members of the Gαi-family (Gαi_1_, Gαi_2_, Gαi_3_, Gαo_a_, Gαo_b_), as well as to Gα12 [120], and several chemokine agonists of CCR2, CCR5 and CCR7 are activated with varying potency that is independent of their affinity for G-proteins, indicating that signaling bias occurs also at this level [120].

**Fig. 7:**
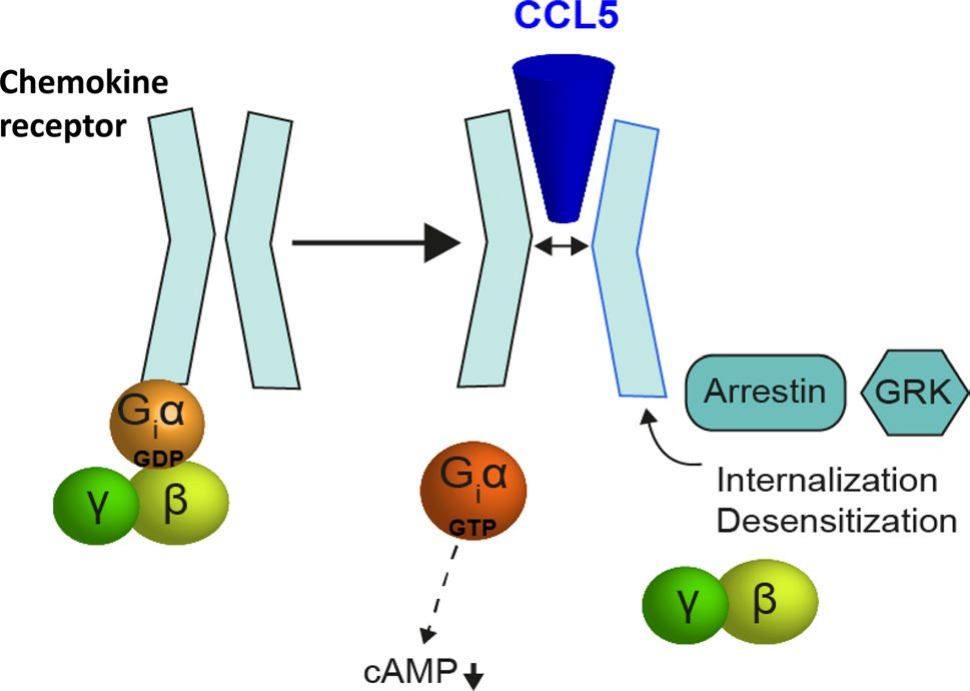
Chemokines bind to receptors, whose cytoplasmic region is an adaptor for G-proteins and can transmit signals via arrestins after their docking following phosphorylation by G protein-coupled receptor kinase (GRK)

This ligand–receptor interaction leads to conformational changes within the Gα state and results in exchange of GDP with GTP (Fig. 7). The heterotrimeric G protein detaches from the receptor, and the βγ- and αi-units separate, thus initiating signaling cascades [119] (Fig. 7). Gαi inhibits adenylate cyclase, and intracellular levels of the second messenger cAMP drop with ensuing effects on the activity of cAMP-dependent protein kinases (PKs). The exact mechanisms as to how Gαi signals induce cytoskeleton rearrangement that drives cell movement are unknown. Distinct signaling pathways and biased signaling are important ways to explain why different ligands (binding as homo- or hetero-dimers to the same GPCR) can have different and even opposing functional effects [121].

Chemokines can also interact and signal via their GPCRs in conjunction with β-arrestins and G protein receptor kinases (GRKs) (Fig. 7). Upon chemokine ligand binding, GPCR cytoplasmic residues become phosphorylated by GRKs, e.g. upon CXCL12 stimulation [122]. Importantly, this type of site-specific phosphorylation of CXCR4 results in both positive and negative modulation of CXCR4-mediated signaling [122]. Phosphorylation at the C-terminus of CXCR4 is structurally distant from the β-arrestin-binding site, yet it is prerequisite for association of β-arrestins with CXCR4 and its internalization. Differences in ligand-binding modes can lead to differences in receptor signaling, a process termed “biased signaling” as reported for the β2-adrenergic receptor [123, 124]. Because the intricacies of signaling can be regulated in a ligand-biased manner, a change in the binding mode, e.g. between CXCL12 and CXCR4 (perhaps caused by heteromerization) can shift or uncouple pathways that lead to chemotaxis, CXCR4 internalization, and CXCR4 desensitization.

To add another layer of complexity, chemokine receptors themselves can oligomerize. Normally, chemokine receptors exist constitutively as dimers, like CXCR4, and/or associate as oligomeric heteromers. By modifying their conformations, chemokines can activate receptors to which they do not primarily bind [125]. E.g. CCR2 forms heterocomplexes with CCR5, and oligomeric complexes of CCR2, CCR5 and CXCR4 also exist [126, 127]. Hetero-oligomerization of chemokine receptors can be induced by binding chemokine heterodimers with functional and therapeutic consequences. Negative and positive cooperative effects can also occur, and the use of specific chemokine inhibitors can result in allosteric *trans*-inhibition [102, 128, 129]. Obviously, the possibility for heterodimer formation facilitates a spatial bridging between different receptors, an activity underlying galectin-mediated lattice formation on the cell surface.

With an eye on molecular interactions among chemokine family members in both homo- and heterodimer states, the scope of similar interactions beyond this family can be extended to tissue lectins with galectins as in situ candidates. This concept warrants a further look into this family of lectins.

## Galectins

Aggregation of erythrocytes (haemagglutination) induced by serum antibodies has revealed the presence of distinct surface epitopes that are the molecular basis of blood groups [130]. Respective typing of red blood cells was also achieved by another type of reagent with an antibody-like level of specificity, i.e. (glyco)proteins in plant extracts termed lectins (from Latin *lectus*, the past principle of *legere* meaning to read, choose or select [131–134]. Sugar-mediated inhibition of lectin-dependent haemagglutination was not only instrumental for identifying carbohydrates as the building blocks for blood group epitopes [135], but established a robust and popular experimental set-up to detect lectin activity.

Because “complex carbohydrate-containing molecules may function in synaptic recognition and transmission through establishment of cell–cell contacts and possibly also as mediators of communication between the surface and the interior of the cell, the presence in neural tissue of enzymes and proteins capable of interacting with saccharides is to be expected” [136]. The testing of extracts from the electric organ of *Electrophorus electricus* (and also chicken, mouse and rat organs and cells) and inhibition of galectin-dependent erythrocyte clumping by β-galactosides (most potently by thiodigalactoside) led to the purification of the first galectin [136]. In this case, the presence of a reducing agent, i.e. dithiothreitol, is essential to preserve activity, leading to coining the first name for such lectins, i.e. S-type lectins. “The finding that S-type lectins form an evolutionarily related family, with certain residues being critically conserved” [137] prompted the search for additional homologous proteins by computer-assisted sequence alignment, and then to progressively track all members of this family at the genome level [138]. In this way, galectins can be viewed in situ as a network of such mediators [139, 140].

## Galectin structures

**Fig. 8:**
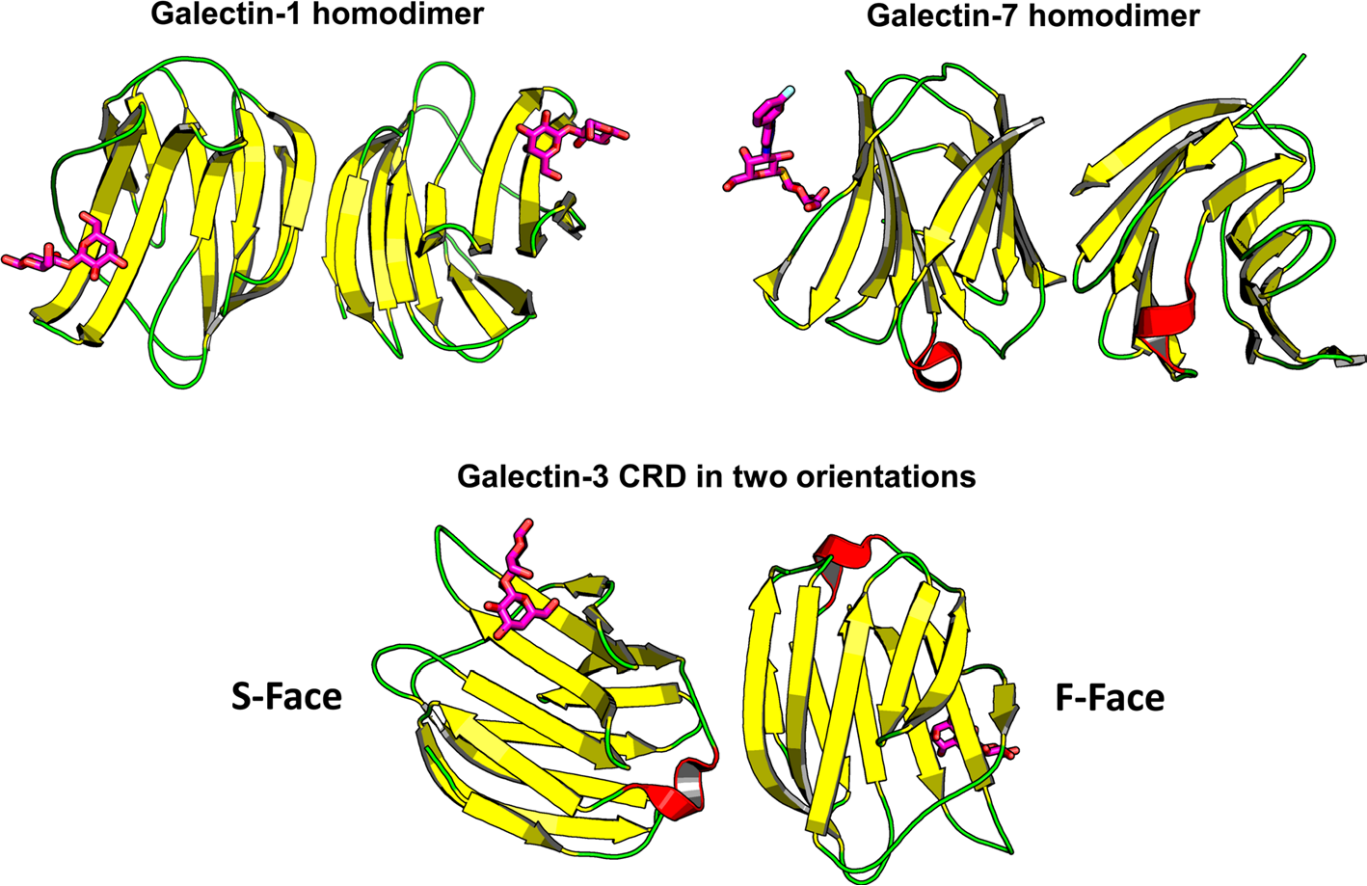
Homodimers of different galectins (Gal-1, PDB code: 1GZW; Gal-3, PDB code 3AYE: Gal-7, PDB code 5H9S). Colors are shown according to their secondary structures (β-strands in yellow, C-terminal α-helix in red, and loops in green), and bound lactose is drawn by magenta sticks. The two β-sheet faces of the CRD β-sandwich (sugar-binding S-face, and opposing F-face with contact site for CXCL12) are indicated with the Gal-3 structure

The common structural feature of galectins is their β-sandwich-type (jelly roll-like) fold of the carbohydrate recognition domain (CRD) as shown in Fig. 8. Intra-family sequence differences among galectins around their β-galactoside binding site defined by a central, highly conserved tryptophan (for C–H/π-interactions with the B-face of galactose) can generally explain differences in ligand-binding affinities [141–143]). Another aspect of functional importance for vertebrate galectins is their quaternary structure in which the CRD can be presented in three types of modular architectures [144, 145]: proto-type (non-covalently associated homodimers, like chemokines), tandem-repeat type (covalently linked heterodimers) and chimera type (natural fusion protein of a CRD with an N-terminal tail for self-association and for post-translational modification by serine phosphorylation). Figure 8 shows the crystal structures of two proto-type galectins (Gal-1 and Gal-7), and the only chimera-type galectin, Gal-3, in which the CRD is devoid of its N-terminal tail has been crystalized. The two β-sheet faces of the conserved CRD β-sandwich structure (sugar-binding S-face, and opposing F-face) are shown for the Gal-3 CRD in Fig. 8, an illustration that underscores the CRD structural bivalency with these two faces.

Even though galectins are known primarily for their recognition of and binding to β-galactosides, they can also interact with other types of glycans at their S-face, e.g. Gal-1 binding to α-linked digalactosides [146] and α-galactomannans [147]. In fact, the sugar-binding domain on the CRD S-face is more extensive for a complex glycan than for simple saccharides which has implications for galectin–glycan interactions at the cell surface [148]. Moreover, Gal-1 and Gal-3 can bind α-galactomannans at an alternative sugar-binding domain on the CRD F-face [147, 149]. These findings greatly broaden the glycospace available to galectins and complicate elucidation of the “sugar code” [137].

The modular arrangement of a given galectin determines the topological properties of galectin–glycoconjugate complexes. This is a salient feature in terms of initiating (or not) cell signaling as inferred from precipitation experiments and negatively stained electron micrographs with glycoclusters and Gal-1 or Gal-3; with Gal-3, rather heterogeneous and disorganized complexes are observed in contrast to well-ordered homogenous Gal-1–glycoconjugate complexes [150]. In cell growth regulation, wild-type galectins with different architectures, i.e. Gal-1 and -3, can thereby be antagonistic [151], and engineered variants with a switch in modular design (i.e. from prototype to chimera type or vice versa for Gal-1 and -3) exhibit different binding profiles in tissues and architecture-dependent responses by inhibiting proliferation or acting as antagonists [25, 152]. These results reveal the functional significance of modular architectures.

## Galectin function

Physiologically, galectin–glycan binding facilitates a broad range of activities, such as intracellular routing, delivery of glycoproteins, and bridging of counter-receptors on the cell surface (in *cis* and *trans*). It is noteworthy that each of these processes is highly specific and attributed to pairing with cell type-dependent counter-receptors [153, 154]. Even a small sugar headgroup, such as that presented by sulfatide, can establish the contact site which makes ‘reading’ possible [155]. For example, long chains of N-acetyllactosamine (polyLacNAc) have high ligand capacity for distinct galectins [156], and for the Gal-3 CRD even allows for clustering with the possibility for stable contacts [157]. With the inflammatory cascade, Gal-8, e.g. can bind glycans of the β_2_-integrin lymphocyte function-associated antigen-1 (LFA-1), an event that allows the lectin to interfere with the integrin’s interaction with intercellular adhesion molecule-1 (ICAM-1) [158]. Although high-resolution structures of a cell receptor-bound galectin are unknown, NMR spectroscopy has been used for insight into the binding of Gal-3 to endothelial cell adhesion molecule (ECAM) CD146 [159].

When functioning intracellularly as sensors for membrane damage, galectins have an intriguing role beyond just glycan binding. For example, Gal-3 and Gal-8 can track membrane rupture that makes complex-type N-glycans accessible via their lectin activity (‘sensing danger’) [27, 28]. Galectins can also ‘call for help’ and organize joint efforts by building molecular bridges from the “danger signal” to components of autophagy or lysosome repair machineries, such as the 52 kDa nuclear dot protein (NDP52) [160] or tripartite motif (TRIM) proteins like TRIM16 [161–163]. With Gal-8 and NDP52, this bridging has been elucidated by crystallographic studies that led to the discovery that hydrophobic interactions and numerous hydrogen bonds between the F-face of the C-terminal CRD of Gal-8 and NDP52 convey specificity to this protein–protein interaction [164, 165]. The canonical glycan-binding site (on the S-face of galectin CRDs) remains open for contact in both CRDs of the tandem-repeat-type Gal-8.

Because galectins have no signal peptide and thus do not enter the ER–Golgi-based export route [166, 167], their cytoplasmic presence predestines them for a role in intracellular surveillance. The case of Gal-8 interacting with an autophagy adaptor (as well as evidence for heterodimer formation among galectins discussed above) on the structural level is the realization of the attractive possibility of hetero-bivalency that enables simultaneous protein–glycan and protein–protein interactions. Following their non-classical secretion from cells (e.g. with Gal-3 in inflammation), galectins will then be exposed to chemokines within the extracellular space, as well as to their receptors and chemokine–receptor complexes on the cell surface in inflamed tissue. Relevant information for the obvious type of binding, i.e. the glycan-dependent interaction, in this setting has been reported, yet so far only in a single instance, i.e. a cytokine and a receptor [168].

On the receptor level, the extent of cellular response to interferon-γ (IFNγ) is sensitive to the frequency of N-glycosylation of the receptor R2 subunit. This effect is linked to Gal-1 and Gal-3 lattice formation that is modulated by acquisition of a new sequon for N-glycosylation by a Thr168 to Asn mutation. In this regard, it shifts the glycoform with an additional N-glycan from sphingolipid/cholesterol to those associated to the actin cytoskeleton, such that the intracellular changes upon ligand binding that activate the JAK/STAT signaling cannot occur [169]. Intriguingly, this N-glycosylated cytokine itself (and also interleukin-12 but not the O-glycosylated chemokine CCL6 and the N-glycosylated CCL1) is a ligand for Gal-3 via glycan binding so that retention of IFNγ in the tumor’s extracellular matrix is made possible by Gal-3 residing there [168]. As conclusion, the glycan-binding S-face of a galectin is active in this aspect of immune activity. Inspired by the ability of chemokines to form heterodimers, we tested the hypothesis that galectin CRDs can also associate as heterodimers via protein–protein interactions.

## Galectin–galectin heterodimers

**Fig. 9:**
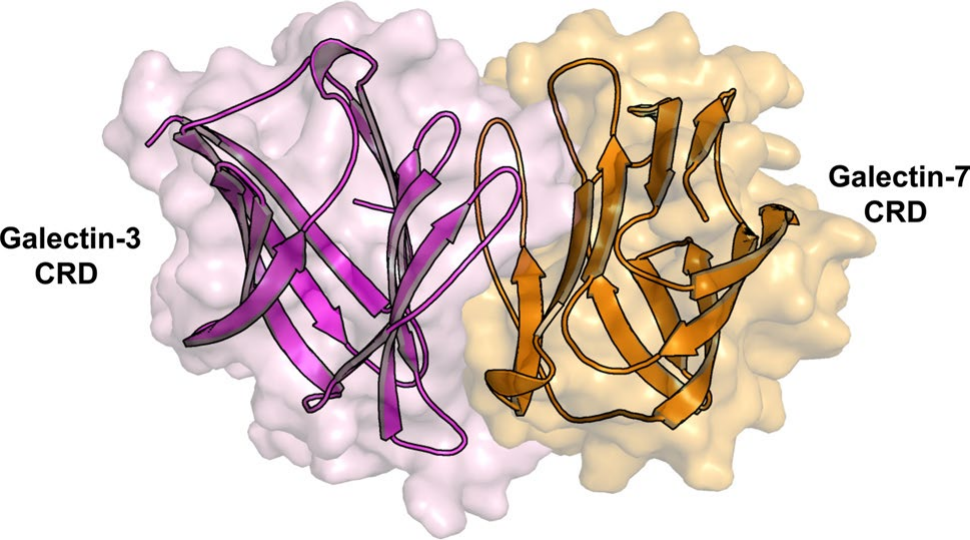
A model is shown for the heterodimer formed between a Gal-3 CRD (magenta ribbon and surface, PDB code: 1AUK) and a Gal-7 CRD (orange ribbon and surface, PDB code: 4GAL). This structural model of the Gal-3/Gal-7 heterodimer was derived by protein–protein docking using NMR data to pinpoint “hot spots” for key interacting residues at the interface, this to guide complex formation [170]. Multiple docking poses were subjected to MD simulations and binding free energy (BFE) calculations. The docking pose represented here exhibited the lowest BFE value, thus indicating the thermodynamically most favorable conformation, which was selected as the most likely structure of the heterodimer

As with chemokines, galectins can indeed form non-covalently associated heterodimers (hybrids) [170]. In this way, the Gal-3 CRD acquires a new level of functionality as a bivalent hybrid where interactions at the heterodimer interface (e.g. those between Gal-3 and Gal-7 CRDs) have been characterized by NMR spectroscopy and molecular modeling (Fig. 9). Intriguingly, *cis/trans*-isomerization of the Pro4 peptide bond in Gal-7 appears to be a molecular switch for the Gal-7 homodimer to dissociate at low concentration, making the Gal-7 CRD available to generate such a hybrid [171]. Cell-binding studies have underscored the possibility of bioactive heterodimers of the Gal-3 CRD with Gal-7 and also Gal-1 CRDs [170]. Environmental conditions can dictate the extent of heterodimerization that extends the bioactivity range of galectins, opening a promising way to look for new, so far unsuspected CRD permutations within the galectin family. Having documented the galectin CRD F-face as a platform for protein–protein contacts, the concept of interplay with a chemokine is not unrealistic.

## Chemokine–galectin heterodimers: example of CXCL12-Gal-3

Collectively, structural data on chemokine–chemokine and galectin–galectin heterodimers provided the incentive to examine the fundamental hypothesis that galectins and chemokines interact to form heterodimers. As outlined below, our first step in this regard was to establish an “interactome” of chemokines that associate with Gal-1 and Gal-3. With NMR resonance assignments and structural characterizations in hand for both galectins [172, 173] and for the chemokine CXCL12 [174, 175], the proof-of-principle was solidly established up to the level of mapping contact sites [176]. This in turn opened the way to explore the potential for this type of interaction and to elicit functional effects in vitro and in vivo, with the perspective of a full-scale network analysis.

As noted above, Gal-1 and -3, like some chemokines, are upregulated during inflammation and are among the most abundant and best studied galectins in inflammatory diseases. Under inflammatory conditions, metalloproteinases cleave the N-terminal region of Gal-3, resulting in free Gal-3 CRD [177–179]. Therefore, the encounter of these galectins with chemokines becomes highly likely and directs our interest to demonstrate heterodimerization by direct interactions in vitro. Solid-phase and surface plasmon resonance-based assays showed that Gal-1 and Gal-3 (as well as the Gal-3 CRD) bind to specific chemokines from CC-, CXC-, XC- and CX3C-families [176]. The K_D_ values for CXCL12 binding to Gal-3 and Gal-3 CRD by surface plasmon resonance are 80 nM and 34 nM, respectively. The chemokine-binding patterns to these galectins demonstrate remarkable selectivity, and, as evidenced by NMR HSQC data analysis, the binding epitope on either galectin does not involve the canonical sugar-binding site on the CRD S-face [176]. Knowledge of the binding epitopes on both the galectin and paired chemokine has allowed us to model the galectin–chemokine heterodimer, a model that was validated by mutagenesis studies [176].

**Fig. 10:**
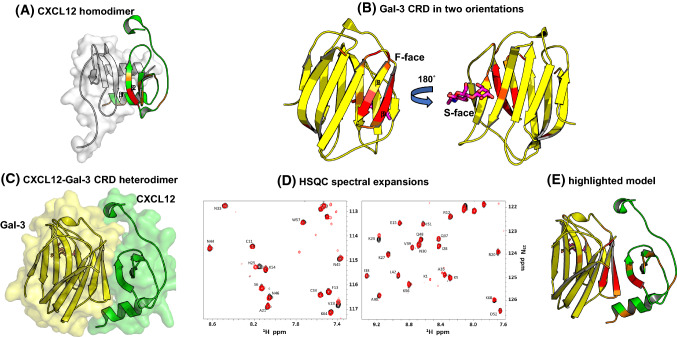
Heterodimer by CXCL12-Gal-3 CRD association. (**A**) The crystal structure of the CXCL12 homodimer (PDB code: 4UAI) is shown with monomer subunits highlighted in green and red. (**B**) The structure of the Gal-3 CRD (in yellow and red) bound with lactose (in magenta) (PDB code: 1A3K) is shown. (**C**) A model of the heterodimer formed between CXCL12 (green) and Gal-3 (yellow) derived from NMR-directed protein–protein docking, MD simulations and BFE calculations is shown [176]. (**D**) Spectral expansions of HSQC data of ^15^N-labelled CXCL12 in the absence (black contours) or presence (red contours) of the unlabelled Gal-3 CRD are shown. (**E**) The NMR-based heterodimer model is shown with residues that are most perturbed by interactions between Gal-3 and CXCL12 highlighted in red and orange

**Fig. 11:**
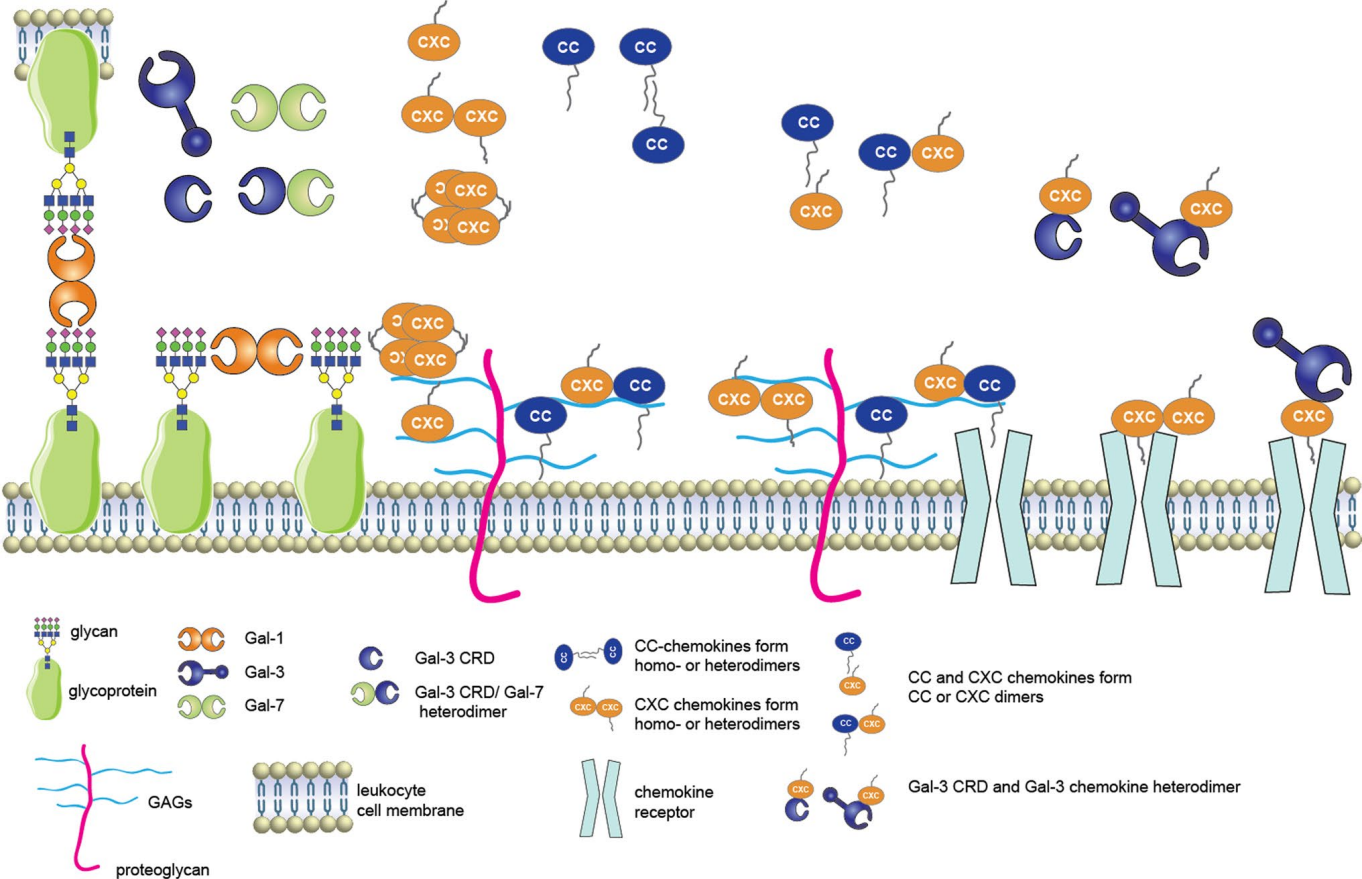
Schematic illustrating possible structural interactions among chemokines and galectins. Chemokines and galectins can be expressed simultaneously in situ, and their function depends generally on which quaternary structure they have. Prototype galectins (e.g. Gal-1 and Gal-7) normally function extracellularly by crosslinking cell surface glycoproteins in a cis (adjacent) or trans (vis-à vis) fashion. The only chimera-type Gal-3 forms heterodimers both with Gal-7 and with chemokines, including CXCL12. Chemokines exist as monomers, dimers and oligomeric forms with CC chemokines forming elongated dimeric structures and CXC chemokines forming more compact dimers. Some chemokines can form heterodimers in a CC- or CXC-type dimeric structure. In the CC-type, the N-termini of two chemokines make up a significant portion of the homodimer interface and can thus not enter the chemokine receptor pocket, whereas the N-terminus remains free in CXC homodimers. Chemokines bind to proteoglycan GAG (glycosaminoglycan) chains as monomers or as oligomers; however, in their monomeric form, they cannot bind to the GAG and also activate their receptor at the same time. CXC chemokines can bind to their receptor as monomers and dimers. Some chemokines bind to galectins, including Gal-3 in its full-length and truncated forms (Gal-3 CRD). Formation of a galectin–chemokine heterodimer can result in a change in chemokine receptor activity. In the case of Gal-3 e.g., its interaction with CXCL12 results in inhibition of CXCR4 and chemotaxis

The largest contiguous Gal-3 CRD contact region with CXCL12 comprises β-strands β8–β9 within the Gal-3 CRD F-face that opposes the canonical sugar-binding S-face of the CRD β-sandwich (Fig. 10). CXCL12 binding to Gal-3 allosterically affects residues within the sugar-binding site, like N160 that is not in direct contact with the CXCL12-binding site on the F-face. This finding has implications for Gal-3’s activity as a lectin, in that Gal-3 in principle can still bind β-galactosides even when CXCL12 is bound. From the perspective of the chemokine, CXCL12’s β1-strand is an important contact site for binding to GAGs, and CXCL12’s binding to heparin attenuates heterodimerization of CXCL12 and Gal-3, an observation that has implications for GAG-regulated biology of CXCL12. In principle, a chemokine dimer can be presented by a GAG and simultaneously to its receptor (Fig. 11) [79]. For the Gal-3-CXCL12 heterodimer, a prerequisite would be that Gal-3 binds to GAGs, and indeed it does [156, 180]. This requires that the dimer interface does not overlap with the GAG/receptor binding site. Our model is based on chemical shifts of CXCL12 when Gal-3 is binding and the affected residues are similar to those of the CXCL12 homodimer indicating an overlap with the receptor binding site and the GAG binding site. Therefore, we believe that a complex of GAG, CXCR4, CXCL12 and Gal-3 cannot exist at the same time. Nevertheless, proteoglycans can play an important role by changing the equilibrium of the individual interactions in which the components come together in close proximity, so that the diffusion distances are short.

Gal-3 (a.k.a. macrophage marker Mac-2 [181]) is expressed by tumor-associated macrophages that are responsible for reducing the mobility of other leukocyte subsets, especially CD8+ T cells [182]. In this respect, the binding of Gal-3 to IFNγ and to chemokines may be mechanistically relevant [168, 176]. Indirectly, Gal-3 inhibits chemokine-dependent migration of CXCR3-expressing lymphocytes by preventing diffusion of IFNγ and in turn the upregulation of chemokines CXCL9, CXCL10, and CXCL11. Recently, evidence for direct biological effects from Gal-3–chemokine heterodimers has been reported with the inhibition of CXCL12- or CCL26-mediated chemotaxis (CXCL12-induced T-cell and neutrophil migration and CCL26-induced eosinophil migration) being attenuated by heterodimer formation with full-length Gal-3 or its CRD [176].

**Fig. 12:**
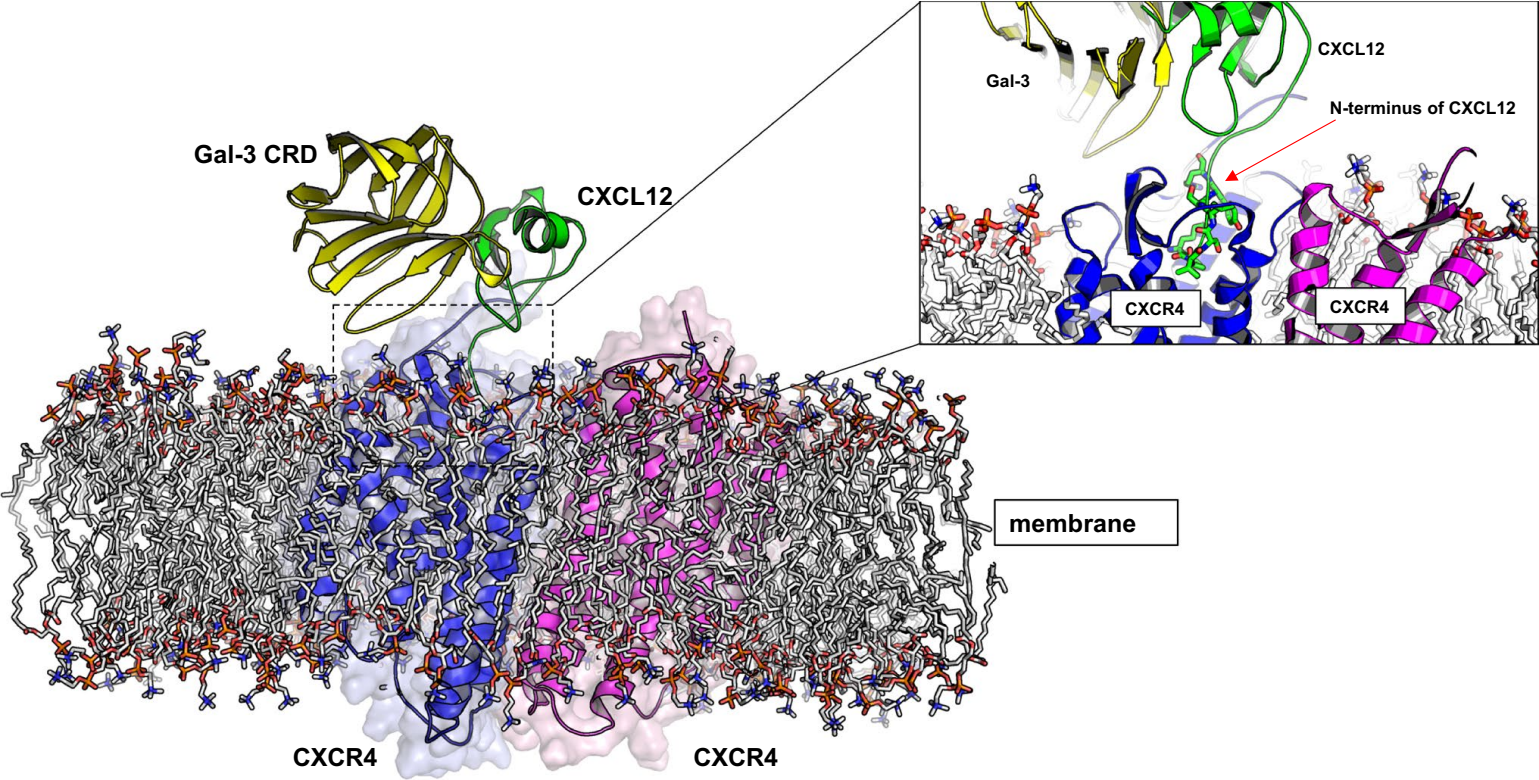
A model of the ternary complex formed between the homodimeric CXCR4 chemokine receptor and the CXCL12–Gal-3 heterodimer. Insert: a closer look of the model reveals that the N-terminus of CXCL12 binds to the CXCR4 receptor without steric hindrance from Gal-3 CRD

With galectin binding to a mediator via a glycan (IFNγ) or protein (CXCL12), inhibition is likely the result of reduced receptor signaling. In the case of IFNγ, it has been proposed that Gal-3 acts as a scavenger for the cytokine [168]. In the case of Gal-3 interacting with CXCL12, heterodimer Gal-3/CXCL12 complexes have been detected on the cell surface, and modeling of the Gal-3/CXCL12 heterodimer bound to CXCR4 shows that bound Gal-3 occurs without steric hindrance of CXCL12 binding to CXCR4 (Fig. 12). These complexes occur at concentrations that are lower than the actual K_D_ values would predict. The reason for this is likely that cell surface structures and molecules, such as GAGs and the glycocalyx, facilitate heteromerization. This model implies that Gal-3 can influence CXCL12 signaling by forming a ternary complex with CXCR4 that induces a conformational change in the GPCR (Fig. 12). Recalling that chemokine heterodimers can induce chemokine receptor heterodimerization, it is possible that a galectin–chemokine heterodimer can bring together a pair of chemokine and galectin receptors. In this regard, formation of chemokine–galectin heterodimers could have wide-ranging biological consequences, opening a new, broad area for study.

## Conclusion

Members from an effector molecule family have usually been analyzed independently of each other, which effectively limits a thorough understanding of their functional versatility and cooperation among members of the group. This approach, in turn, can miss physiologically important possibilities for “teamwork” among protein effectors from different classes by specific associations (i.e. hybrid formation) when they occur within the same micro-environment. Relatedly, the following points favor broadening investigations into our heterodimer discoveries: (i) concentrations of some chemokines and galectins are elevated at sites of inflammation, (ii) chemokines and galectins can interact with each other within their family in diverse manners, including heterodimer formation, and (iii) galectins can engage in glycan and protein recognition with chemokines. The CXCL12–Gal-3 interaction studied on the structural level by NMR spectroscopy and molecular modeling provides a proof-of-concept for specific pairing of galectins and chemokines. This, in turn, provides direction to systematic structural and functional analyses of chemokine–galectin heterodimers. The engineering of stable heterodimers (termed lectinology 4.0 [183] for galectins) would enhance the emerging physiological significance of these new classes of molecular hybrids.

## Data Availability

Not applicable.
